# WHO multicentre study for the development of growth standards from fetal life to childhood: the fetal component

**DOI:** 10.1186/1471-2393-14-157

**Published:** 2014-05-02

**Authors:** Mario Merialdi, Mariana Widmer, Ahmet Metin Gülmezoglu, Hany Abdel-Aleem, George Bega, Alexandra Benachi, Guillermo Carroli, Jose Guilherme Cecatti, Anke Diemert, Rogelio Gonzalez, Kurt Hecher, Lisa N Jensen, Synnøve L Johnsen, Torvid Kiserud, Alka Kriplani, Pisake Lumbiganon, Ann Tabor, Sameera A Talegawkar, Antoinette Tshefu, Daniel Wojdyla, Lawrence Platt

**Affiliations:** 1 UNDP/UNFPA/UNICEF/WHO/WORLD BANK Special Programme of Research, Development and Research Training in Human Reproduction; Department of Reproductive Health and Research, World Health Organization, Geneva, Switzerland; 2 Assiut University, Assiut, Egypt; 3 Thomas Jefferson University, Philadelphia, PA, USA; 4 Hôpital Antoine Béclère, Université Paris Sud, Paris, France; 5 Centro Rosarino de Estudios Perinatales, Rosario, Argentina; 6 University of Campinas, Campinas, Brazil; 7 University Medical Center, Hamburg, Germany; 8 Pontificia Universidad Católica de Chile, Santiago, Chile; 9 University of Bergen, Bergen, Norway; 10 All India Institute of Medical of Sciences, New Delhi, India; 11 Khon Kaen University, Khon Kaen, Thailand; 12 Copenhagen University Hospital Rigshospitalet, Copenhagen, Denmark; 13 Johns Hopkins Bloomberg School of Public Health, Baltimore, USA; 14 University of Kinshasa, Kinshasa, Democratic Republic of Congo; 15 Center for Fetal Medicine, Los Angeles, USA

**Keywords:** Fetal growth, Child development, Ultrasound, Growth standards

## Abstract

**Background:**

In 2006 WHO presented the infant and child growth charts suggested for universal application. However, major determinants for perinatal outcomes and postnatal growth are laid down during antenatal development. Accordingly, monitoring fetal growth *in utero* by ultrasonography is important both for clinical and scientific reasons. The currently used fetal growth references are derived mainly from North American and European population and may be inappropriate for international use, given possible variances in the growth rates of fetuses from different ethnic population groups. WHO has, therefore, made it a high priority to establish charts of optimal fetal growth that can be recommended worldwide.

**Methods:**

This is a multi-national study for the development of fetal growth standards for international application by assessing fetal growth in populations of different ethnic and geographic backgrounds. The study will select pregnant women of high-middle socioeconomic status with no obvious environmental constraints on growth (adequate nutritional status, non-smoking), and normal pregnancy history with no complications likely to affect fetal growth. The study will be conducted in centres from ten developing and industrialized countries: Argentina, Brazil, Democratic Republic of Congo, Denmark, Egypt, France, Germany, India, Norway, and Thailand. At each centre, 140 pregnant women will be recruited between 8 + 0 and 12 + 6 weeks of gestation. Subsequently, visits for fetal biometry will be scheduled at 14, 18, 24, 28, 32, 36, and 40 weeks (+/− 1 week) to be performed by trained ultrasonographers.

The main outcome of the proposed study will be the development of fetal growth standards (either global or population specific) for international applications.

**Discussion:**

The data from this study will be incorporated into obstetric practice and national health policies at country level in coordination with the activities presently conducted by WHO to implement the use of the Child Growth Standards.

## Background

Of the estimated 4 million neonatal deaths each year, more than 60% are associated with low-birth-weight due to intrauterine growth restriction and/or preterm delivery [1]. Accurate prenatal assessment of fetal growth and gestational age to timely identify and adequately manage cases of growth restriction and/or preterm delivery should be considered a public health priority, especially in developing countries where 98% of the worldwide neonatal deaths occur.

Before the advent of ultrasonography, fetal growth was assessed by evaluating newborn weight as the end point of the intrauterine growth process. However, cumulative evidence indicated that this birth weight curve approach did not accurately describe the fetal growth process for newborns born before 37 weeks of pregnancy, and low birth weight babies born at term. In response to this evidence, the prenatal assessment of fetal growth and gestational age *in utero* by means of ultrasonography was adopted as standard practice in prenatal care throughout the world. The adequacy of fetal size is currently assessed by comparing the measurements of fetal anatomical parameters at a given gestational age captured through ultrasonography with reference percentiles of fetal size derived from populations of fetuses whose growth was assumed to be normal. These same reference percentiles can be used to estimate gestational age from observed fetal size. The most commonly used reference charts of size by gestational age were developed based on data from populations of fetuses in the United States or Europe. Since 1981, concerns have been raised that such charts might not be appropriate for use in other ethnic groups which may experience different patterns of fetal growth [2-4]. If fetal growth is dependent upon ethnic heritage, the potential for misclassification of fetal growth abnormalities through the wide-scale application of existing growth reference charts should generate concerns regarding diagnostic and management decisions made on the basis of ultrasonographic fetal growth assessments. Others counter argue that environmental factors play a more critical role in fetal growth than ethnicity, and that all fetuses should undergo comparable growth patterns in the absence of environmental constraints to growth [5]. The need to resolve this debate makes the development of fetal growth standards for international application both timely and a priority for improving maternal, fetal and newborn heath care worldwide [6].

Reliable standards of fetal growth are important for assessing the wellbeing of each maternal-infant dyad, determining the health status of populations, and monitoring progress in fetal and newborn health and development. Accurate fetal growth assessment is also important for maternal health. Fetal growth abnormalities are often associated with pregnancy complications such as hypertensive disorders and may affect how specific pregnancies are managed (e.g. the decision to perform a caesarean section). Thus, the results of this study will be beneficial for maternal and fetal/newborn health, they will support efforts to provide a continuum of care for mothers and their infants, and will be relevant to country efforts to accelerate progress to achieving Millennium Development Goals 1, 4, and 5.

The following sections briefly describe the work done and recommendations issued by the World Health Organization in the field of growth assessment that led to the development of this proposal.

In 2006 WHO released the WHO Child Growth Standards. These curves were the result of the WHO Multicentre Growth Reference Study (MGRS) [7] which had two characteristics that made the study unique in its field: i) the prescriptive approach [8-10]; ii) the international representation.

The proposed study is an extension of the MGRS to fetal life. It will be based on the same prescriptive approach for sample selection, and will ensure international representation by including populations from five continents. The main outcome of the study, fetal growth reference standards, will complement the MGRS by extending the WHO Child Growth Reference Charts to the prenatal period.

In 2002, a meeting of experts on the life course and health convened by the WHO Department of Non Communicable Diseases and Mental Health, Chronic Diseases and Health Promotion, identified as a top research priority, the “improvement of measures of intra-uterine growth retardation (alternative to low-birth-weight) which must reflect newborn body composition and fetal exposures that may not necessarily be expressed in birth size” [11]. Recent findings relating fetal femur length as assessed in-utero by ultrasonography with blood pressure levels in childhood confirm that measures of fetal growth alternative to birth weight provide important information which may help clarify the potential associations between fetal growth abnormalities and postnatal risk of disease [12].

In December 2002, the WHO Department of Nutrition organized a meeting of experts to review current knowledge and the practical implications of the interpretation of birth weight as a health outcome. Further research to develop fetal growth standards was identified as urgent by the group.

Finally, in 1995, the WHO Expert Committee on Physical Status published the results of a three year collaborative effort involving more than 100 experts who reviewed the currently available data on body size and composition at the different stages of life and their interpretation in terms of nutrient intake, activity level and risk of disease [13,14]. As a follow up of these recommendations, WHO implemented and conducted the MGRS. In addition, the WHO Expert Committees also recommended the development of fetal growth reference data suitable for international applications [5]. The proposed study intends to implement those recommendations as a logical extension of the MGRS.

## Methods

### Study design

This will be a multi-country observational study aiming at developing fetal growth standards for international application. This study will use an inclusive approach when selecting participating centres so that diverse ethnic population groups and diverse geographic settings are adequately represented. The following centres have been identified to participate in the study based on the proficient use of ultrasonography:

•Argentina: Centro Rosarino de Estudios Perinatales, Rosario

•Brazil: University of Campinas, Campinas

•Democratic Republic of Congo: University of Kinshasa, Kinshasa

•Denmark: Copenhagen University Rigshospitalet, Copenhagen

•Egypt: Assiut University, Assiut

•France: Hôpital Antoine Béclère, Paris

•Germany: University Medical Center, Hamburg

•India: All India Institute of Medical of Sciences, New Delhi

•Norway: University of Bergen, Bergen

•Thailand: Khon Kaen University, Khon Kaen

### Eligibility criteria

In terms of the selection of a study participant, the protocol will follow a prescriptive approach. Participants with no known health, environmental, and/or socio-economic constraints on fetal growth will be invited to participate in the study if:

•They belong to a high socio-economic status and a high level of parental education, in order to ensure that the curves reflect, as much as possible, the true growth potential of fetuses, by limiting, as much as possible, the influence of environmental factors. Specific cut off points in family income and education have been identified by the MGRS and will be translated into socio-economic indicators specific to the countries participating in the proposed study [9,10,15];

•They live at an altitude lower than 1,500 m;

•They live near the study area. This will ensure compliance with the study, follow up for the study duration, and for potential future follow-up studies in infancy, childhood and eventually adulthood;

•They are 18 years old or more (as younger women are still growing and their babies may be smaller at birth) and ≤ 40 years;

•They have a BMI between 18–30;

•They have a singleton pregnancy;

•Their gestational age at entry is between 8 + 0 to 12 + 6 weeks based on LMP (confirmed by ultrasonography, please see Dating by ultrasound for a more detailed description);

•They have no history of health, environmental or economic constraints likely to impede fetal growth; no need for long-term medication (including fertility treatment); not smoking currently or in the previous 6 months; no history of recurrent miscarriages; and any baby previously delivered pre-term (<37 weeks) or with a birth weight <2,500 g (at 37 w 5% of boys and 10% of girls in low-risk pregnancies will be ≤2,500 g);

•There is no evidence in the present pregnancy of congenital disease or fetal anomaly. Participation in the study will cease if a major fetal anomaly is detected or serious illness develops leading to IUGR (birth weight below the 10th percentile of the recommended gender-specific birth weight for gestational age reference curves [16]); however, all mothers recruited will be followed-up until the end of the study for the purposes of describing the whole population.

### Study procedure

Women in the first trimester (before 12 + 6 weeks gestation) attending antenatal care clinics providing ultrasonographic examinations will be approached by members of the study team and asked to participate. Women will be fully informed about the study objectives and procedures. Only women who sign a consent form will be enrolled into the study.

Fetuses will be scanned in the first trimester for the estimation of gestational age and subsequently at monthly intervals for fetal biometry [17].

All infants will receive an anthropometrical assessment after delivery, including measurement of birth weight [18]. All pregnant women in the study will be administered a 24-hour dietary recall at entry into the study, and at approximately 28 and 36 weeks gestation, to assess maternal nutritional status and ensure that women enrolled in the study have an adequate diet and that nutrient intake is in accordance with current pregnancy recommendations [18,19]. At each visit, the obstetric history of participating women will be updated to collect information related to pathological processes that may affect fetal growth, and blood pressure and proteinuria will be measured.

No additional procedures will be added to routine antenatal care provided at the study centres, with the exception of 4–5 additional ultrasonographic examinations and three repeat 24-dietary recalls.

### Dating by ultrasound

Gestational age will be confirmed by measuring the crown-rump length (CRL) between 8 + 0 to 12 + 6 weeks based on LMP. Gestational age (GA) by CRL should agree with GA by LMP to within 7 days. If LMP and CRL agree, CRL measurement will be used for dating. The average of three measurements will be used. If GA by CRL and GA by LMP differ by more than 7 days, the woman is not eligible for the study.

To acquire the CRL measurements, the midline sagittal section of the whole fetus will be visualized with the fetus horizontal on the screen at 90 degrees to the angle of insonation [20]. GA will be assessed by using the reference charts published by Robinson and Fleming [19].

### Fetal biometry

The first visit (dating scan) will be between 8 + 0 and 12 + 6 weeks, and subsequent visits for fetal biometry will be scheduled at approximately 4 weekly (+/− 1 week) intervals at 14, 18, 24, 28, 32, 36, and 40 weeks. All scanning appointments will be arranged at the time of the dating scan and study enrolment. All participants will be scanned in the lateral recumbent position.

The compulsory ultrasound measurements to be obtained at all visits include the following biometrical parameters:

•Biparietal diameter (BPD)

•Head circumference (HC)

•Abdominal circumference (AC)

•Femur length (FL)

•Transcerebellar diameter (TCD)

•Humerus length (HL)

•Fetal Foot length (FFL)

At each examination, all measurements are to be obtained three times from three separately generated ultrasound images and uploaded electronically (with the associated images) to the data management system. The mean of the three measurements of each parameter will be used for clinical management purposes as per local protocols.

In addition, a full morphological evaluation (abnormality scan) will be conducted at 18–24 weeks following standard practices at each centre. Fetuses diagnosed with any minor abnormalities will be managed according to local clinical guidelines. If the clinical decision is to continue with the pregnancy the case will remain in the study. Fetuses with major abnormalities that may affect morphometric measurements will be excluded from further study. All infants will receive an anthropometrical assessment after delivery [21].

The following measurement techniques will be used:

•Biparietal Diameter-Technique: Measured from the outer-outer (BPD 1) and outer – inner (BPD 2) edges of the parietal bones in a cross-sectional view of the fetal head at the level of the thalami and cavum septum pellucidum or cerebral peduncles. The cerebellum is not to be included. The measurement should be obtained from an image with the midline echo as close as possible to the horizontal plane with the angle of insonation of the ultrasound beam at 90 degrees.

•Head Circumference -Technique: Obtained from the same image as BPD as follows: Measurement of occipito-frontal diameter (OFD) obtained by placing calipers on the outer borders of the occipital and frontal edges of the skull at the point of the midline across the longest part of the skull. The ellipse facility will be used to calculate HC as above.

•Abdominal Circumference - Technique: The sonographer will visualize the transverse section of the fetal abdomen as “close as possible” to circular including the stomach and the junction of the umbilical vein and portal sinus. The anterior-posterior (A-P) and transverse diameters will be measured with calipers placed on the outer borders of the body outline. The A-P diameter will be measured from the spine to the anterior abdominal wall and transverse diameter at a right angle to the A-P diameter. The ellipse facility will be used to calculate AC as outlined above.

•FL-Technique: Measured from an image of the full femoral shaft in a plane as close as possible to a right angle to the ultrasound beam. The distal femoral epiphysis is to be excluded.

•Transcerebellar Diameter (TCD): the TCD can be imaged from the sub-occipito-bregmatic view of the fetal skull, and measured from the second trimester onwards. The calipers will be placed on the outer-outer margins of the cerebellar poles.

•Humerus length (HL): Measured from an image of the full humeral shaft in a plane as close as possible to a right angle of insonation.

•Fetal Foot Length (FFL): Fetal foot length will be measured from the second trimester onwards. The foot is measured from either sagittal or plantar views. The measurement is taken from the skin overlying the heel (calcaneus) to the end of the longest toe.

### Ultrasound volume acquisition protocol

The ultrasonographic equipment that will be used in the study will allow for acquiring and storing 3-dimensional images. This feature is of critical importance for data quality purposes. Stored 3-dimensional images (volumes) could be used at a later stage to retake measurements that have been identified as erroneous.

Except for a transvaginal scan during the first research visit (8 0/7 -12 6/7 weeks), 3D volume data will otherwise be acquired using a transabdominal probe. All volumes shall be systematically labelled using the comment feature of the Voluson E8 (GE Healthcare, Germany) Expert system (e.g. “LA1”).

Recommended Image settings:

•At least High Quality 2

•CRI no greater than 2

•No speckle reduction (SRI)

•Harmonic imaging as needed

•Acoustic focus adjusted for the anatomic region

•Use the widest image window to capture the volume of interest, depending on fetal activity

•Adjust the magnification and image depth settings to fill at least one-half of the screen

### Training

As done in previous studies [3,22] sonographers participating in the study will receive specific training and will be certified as proficient under the supervision of a qualified instructor, according to a standard protocol. Intra-observer and inter-observer measurement errors will be assessed according to a published protocol before the initiation of the study during the training period [21]. We will consider estimating a learning course [23].

All instruments and techniques to be used in all centres will be standardised, i.e. equipment and training will be provided to each of the measurement teams. Equipment specifications to be considered are the following: i) Commercially available high quality real-time ultrasound scanner; ii) Less than 2 years old; iii) T/V and abdominal probes suitable for scanning throughout pregnancy; iv) Facility for on-line transfer of measurements and associated images; v) Facility to “blind” measurements from examiner until after data transfer.

Site visits to the study centres will be organized in order to provide lectures and update courses by experts in the field. In addition during site visits, standardization sessions will be carried out according to repeat-measure protocols to assess the accuracy and precision of the anthropometrical measurements in mothers and newborns.

### Neonatal anthropometrical assessment

Neonatal body composition assessment during the first 24 hours will be used to determine the growth outcome of each pregnancy on the basis of multiple postnatal measurements in order to be able to relate pre- and postnatal anthropometrical measurements. After delivery, a trained investigator will take standardized measurements of the head circumference, abdominal circumference (superior border of umbilicus), and thigh circumference (at the skin crease located midway between the knee and trunk with the lower leg at about 90 degrees in relation to the thigh measured in centimetres) with non-stretchable tape. The crown-heel length will be obtained by placing the supine infant, with extended legs, on a plastic newborn length board (Statiometer, Ellard Instrumentation, Seattle, WA, USA). Skin fold caliper measurements (Harpenden) will be used to document soft tissue distribution [17,24]. These skin folds will include the triceps fold, anterior thigh fold, sub-scapular skin fold, and abdominal flank skin fold that will be made at each site twice and averaged. This data will be used to estimate per cent body fat and lean body mass as an index of neonatal growth outcome. Other parameters of neonatal body composition will include ponderal index and birth weight [25].

### Nutritional assessment

Adequate nutrition is one of the major requirements for selecting populations eligible for the study. Therefore, the assessment and maintenance of adequate nutritional status is considered a critical component of the study activities.

At three times during the study follow-up (at entry and at approximately 28 and 36 weeks) the nurse/nutritionist, trained in collecting anthropometric and diet data, will assess maternal nutritional status *via* anthropometry (weight, height, arm circumference, head circumference, skinfolds) (Table 1), as well as assessing the dietary intake (by twenty-four-hour recall). Measurements will be carried out by a female nurse/nutritionist in a private room according to the procedures described by Gibson [17]. The time of the examination will be recorded to allow for diurnal variations. Having only one person performing the measurements will minimize inter-examiner errors. The equipment required is already available at the study site.

**Table 1:** Description of maternal anthropometric measures

**Measure**	**Procedure**
Weight	Weight will be measured using a beam balance with nondetachable weights. Weight will be recorded to the nearest 0.1 kg [26].
Height	Height will be measured in the standing position using a stadiometer and recorded to the nearest millimetre. If the reading falls between two values, the lowest millimetre will be recorded [26].
Mid-upper arm circumference	Measurements will be taken using a flexible fiberglass tape wrapped around the upper left arm, at the midpoint between the acromion process and the tip of the olecranon. Measurements will be recorded to the nearest millimetre [26].
Head circumference	A flexible fiberglass tape will be used. The tape will be placed above the supra-orbital ridges and over the part of the occiput which gives the maximum circumference. Measurements will be taken to the nearest millimetre [26].
Skinfolds	Left triceps and left scapular skinfolds will be taken using a Lange skinfold thickness caliper. The combination of one body and one limb skinfold to assess body fat is recommended by most investigators [26].

Dietary intakes will be assessed using 24-hour recalls; specifically a trained nutritionist or nurse will query the study participants on foods and beverages consumed by them in the previous 24-hours. Information will also be collected on portion sizes as well as preparation methods. This information will then be linked with appropriate country-specific food composition tables to arrive at estimates of macro and micro-nutrients [27,28]. This method results in the attainment of estimates of the intake of single nutrients. Compliance is high because the respondent burden is low. The interview takes approximately twenty minutes. The quality of the information collected is dependent on the respondent’s motivation and ability to recall intakes and on the interviewing skills of the nutritionist/nurse. Repeated twenty-four-hour recalls on the same individual allow estimations of the individual’s usual dietary intake over a long period of time [28]. In our study the twenty-four-hour recall will be repeated three times for each woman. This method has been successfully used in previous studies focusing on nutrition, fetal growth and other pregnancy outcomes conducted by investigators who are taking part in the proposed study.

### Safety of ultrasonographic assessment

Ultrasonography in pregnancy is considered a safe procedure and in more than 30 years no fetal harm has been reported with use in the low-intensity range of gray-scale imaging (no Doppler), which is the technology that will be used in the proposed study [29]. Serial ultrasonography for research purposes according to schedules similar to the one we propose has been approved previously in the context of other studies [30-33].

Following a recommendation of the WHO Scientific and Ethical Review Group, we conducted a systematic review and meta-analysis to evaluate the safety of human intrauterine exposure to ultrasonography [32].

We systematically searched electronic databases, reference lists and unpublished literature according to the following criteria:

•Types of studies: Trials and observational studies that assessed short and long term effects of exposure to ultrasonography during pregnancy.

•Types of participants: Women submitted to ultrasonography in pregnancy and their offspring.

•Types of exposure: B-mode or Doppler sonography during any period of pregnancy, for any number of times, using any equipment and transducers.

•Types of outcome measures: 1) adverse maternal outcomes, 2) adverse perinatal outcomes, 3) abnormal childhood neurological development, and 4) childhood malignancies.

The electronic search identified 6716 citations and 63 were selected for full text evaluation. Additionally, 19 citations were identified from secondary sources. A total of 58 references reporting data of 38 different studies were included: 16 clinical trials, 11 cohorts, and 11 case controls. Ultrasonography in pregnancy was not associated with adverse maternal effects, impaired physical or neurological development or increased risk for malignancies in childhood. According to the clinical trials, there was a weak association between exposure to ultrasonography and non-right handedness in boys (OR 1.26, 95% CI 1.03-1.54) and a slight decrease in mean neonatal length (WMD −0.26 cm, CI −0.45, −0.07) and head circumference (WMD −0.15 cm, CI-0.29, −0.01). In conclusion, based on the available evidence, exposure to diagnostic ultrasonography during pregnancy appears to be safe.

### Sample size

The total sample size will be 1400 pregnant women and their infants. Each country centre will collect data from 140 women. This number is sufficient for the development of local centile growth charts with a high level of precision, accounting for exclusions to final analysis due to inability to follow up and occurrence of pregnancy complications [34,35].

The sample size needed for the estimation of a specific percentile was computed using the following formula [36]:

$$n=\frac{\left(1+z_{\alpha }^{2}/2\right)}{{\left(\frac{\%\mathrm{SE}\;P_{\alpha }}{\%\mathrm{CV}}\right)}^{2}}$$

where z_α_ is the standard normal deviation corresponding to the percentile being estimated, % SE C_α_ is the expected percentage standard error of the percentile and % CV is the percentage coefficient of variation. Sample sizes were computed for 5 different parameters (biparietal diameter, abdominal and head circumference and femur and humerus length) and for 3 different percentiles: 5%, 10% and 50%. Information about the coefficient of variation was obtained from data on serial ultrasonographic examination conducted on approximately 500 pregnancies in the context of the WHO randomized trial of calcium supplementation in low intake women [37]. Additionally, the expected half-width of the confidence interval with a sample size n = 100 was computed.

Table 2 shows the levels of precision obtainable with a sample size of 100 women for various biometrical parameters. In addition the proposed sample size of 140 women per centre will allow for testing for differences in growth patterns across centres [38].

**Table 2:** Levels of precision obtained with a sample size of 100 women for some biometrical fetal parameters (values and half widths in mm)

**Abdominal circumference**										
Week	Mean	10% Centile	5% Centile	CV	%SE_50	%SE_10	%SE_5	Width_50	Width_10	Width_5
20	147.7	133.5	128.8	7.9	0.79	1.07	1.21	2.33	2.85	3.12
24	190.7	173.8	166.6	7.4	0.74	1.00	1.14	2.82	3.47	3.78
28	235.2	215.4	210.6	6.4	0.64	0.86	0.98	3.01	3.72	4.13
32	278.6	255.8	248.3	6.5	0.65	0.88	1.00	3.62	4.49	4.95
36	314.5	288.0	277.9	7.1	0.71	0.96	1.09	4.47	5.52	6.05
**Biparietal diameter**										
Week	Mean	10% Centile	5% Centile	CV	%SE_50	%SE_10	%SE_5	Width_50	Width_10	Width_5
20	46.7	44.0	42.0	5.6	0.56	0.76	0.86	0.52	0.67	0.72
24	59.0	56.0	55.0	4.8	0.48	0.65	0.74	0.57	0.73	0.81
28	70.8	68.0	67.0	3.9	0.39	0.53	0.60	0.55	0.72	0.80
32	80.1	77.0	75.0	3.7	0.37	0.50	0.57	0.59	0.77	0.85
36	87.1	83.0	81.0	3.7	0.37	0.50	0.57	0.64	0.83	0.92
**Head circumference**										
Week	Mean	10% Centile	5% Centile	CV	%SE_50	%SE_10	%SE_5	Width_50	Width_10	Width_5
20	177.4	164.4	161.2	5.8	0.58	0.78	0.89	2.06	2.57	2.87
24	225.0	212.3	209.2	4.9	0.49	0.66	0.75	2.21	2.81	3.14
28	269.4	254.9	251.8	4.3	0.43	0.58	0.66	2.32	2.96	3.32
32	302.8	286.9	281.6	4.1	0.41	0.55	0.63	2.48	3.17	3.54
36	325.1	308.1	300.1	4.1	0.41	0.55	0.63	2.67	3.41	3.77
**Femur length**										
Week	Mean	10% Centile	5% Centile	CV	%SE_50	%SE_10	%SE_5	Width_50	Width_10	Width_5
20	32.6	30.0	29.0	6.9	0.69	0.93	1.06	0.45	0.56	0.61
24	43.2	41.0	40.0	4.7	0.47	0.63	0.72	0.41	0.52	0.58
28	53.0	51.0	50.0	3.7	0.37	0.50	0.57	0.39	0.51	0.57
32	61.9	59.0	58.0	3.3	0.33	0.45	0.51	0.41	0.53	0.59
36	69.3	67.0	65.0	3.2	0.32	0.43	0.49	0.44	0.58	0.64
**Humerus length**										
Week	Mean	10% Centile	5% Centile	CV	%SE_50	%SE_10	%SE_5	Width_50	Width_10	Width_5
20	31.4	28.0	27.0	8.9	0.89	1.20	1.37	0.56	0.67	0.74
24	40.7	38.0	37.0	6.8	0.68	0.92	1.04	0.55	0.70	0.77
28	48.6	45.0	44.0	6.3	0.63	0.85	0.97	0.61	0.77	0.85
32	55.7	52.0	51.0	5.7	0.57	0.77	0.87	0.63	0.80	0.89
36	61.2	57.0	56.0	5.4	0.54	0.73	0.83	0.66	0.83	0.93

The following table lists, for every biometrical parameter, the number of subjects needed to detect as statistically significant the smallest meaningful difference d with type I error rate of 0.05 and a power of 0.80 with values of variance (σ^2^) and correlation (ρ^2^) as estimated using the data from the WHO randomized trial of calcium supplementation in low intake women [37]. The presented sample sizes have been calculated by applying the formula:

$$m=\frac{2{\left(z_{\alpha }+z_{Q}\right)}^{2}\sigma^{2}\left(1-\rho \right)}{ns_{x}^{2}d^{2}}$$

Where d is the minimum difference in the rate of change per unit time (week) in the five biometrical parameters by centre, n is the number of visits (n = 8) and $s_{x}^{2}$ is the within subject variance [38]. The values of d shown in Table 3 indicate that the sample size needed for the level of precision requested will be very conservative to detect differences in the rate of change between centres.

**Table 3:** Estimated sample size and computation assumptions for biometrical parameters

**Biometrical parameter**	**Sample size**	**Variance**	**Rho**	**d (mm)**
Biparietal Diameter	32	10.2	0.34	0.1
Head circumference	23	174	0.28	0.5
Abdominal circumference	28	263	0.43	0.5
Femur length	16	5	0.32	0.1
Humerus length	35	11	0.32	0.1

### Data management

Data will be collected *via* an internet based data management system as done with other WHO coordinated studies. The online data collection system will allow for real time solutions of queries or other problems in data collection, such as missing or non-valid data as well as for online checking of images by the international coordination unit.

All data will be stored in a GCP compliant server, and data transmission will be encrypted to assure data integrity and patient confidentiality. Access to the data management web system will be password protected and only authorized users will have access. Data changes will be documented. The system will maintain an audit trail, data trail and edit trail as well as back up of the data.

Data entered into the web system will be checked by the coordinating unit at WHO for completeness, accuracy, reliability and consistent intended performance. The data management team will be responsible for generating the interim and final data report.

These procedures have been used in previous HRP multi-centre trials and proven to be efficient and compliant with the HRP/WHO Standard Operating Procedures as well as with the 21 CFR Part 11 of the Code of Federal Regulations that deals with the United States Food and Drug Administration (FDA) guidelines on electronic records.

### Data analysis

Population percentiles will be calculated by applying polynomial regression methods, using the generalized estimating equations method, to model the mean at specific gestational ages, taking into account correlations between repeated measurements on the same subjects [38]. To estimate the standard deviations at each gestational age, we will use the method of the absolute residuals proposed by Altman [39]. This methodology takes into account the increase in variance with advancing gestation typical of fetal biometry data [39]. Differences in linear growth among populations of different geographical origin (categorized by study site) will be tested by evaluating the proportion of total variability in fetal biometrical measurements attributable to sites and individuals, as well as differences among sites and the effect of excluding sites on the percentiles of the total sample, as done in the MGRS [40]. The same procedure will be used to test for differences in growth related to the sex of the fetus.

## Discussion

The data from this study, if findings are similar across diverse populations, may facilitate the adoption of an universal growth standard for international use. If heterogeneity by centre is detected despite selecting study populations according to the prescriptive approach, the study results will indicate the need to develop local/ethnically specific standards.

### Technical and ethical approvals

The protocol received technical and ethical approval from the WHO Research Project Review Panel (RP2) and Research Ethics Review Committee respectively as well as from appropriate national and institutional ethic review bodies as applicable for each study centre.

## Abbreviations

AC: Abdominal circumference; BMI: Body mass index; BPD: Biparietal diameter; CH: Chest; CRL: Crown rump length; CV: Coefficient of variation; EV: Endovaginal; FFL: Fetal foot length; FL: Femur length; GA: Gestational age; H: Head; HC: Head circumference; HL: Humerus length; ICC: Intraclass correlation coefficient; ISUOG: International Society for Ultrasound in Obstetrics and Gynaecology; IUGR: Intra uterine growth restriction; LA: Lower arm; LL: Lower leg; LMP: Last menstrual period; MDG: Millennium Development Goal; MGRS: Multi-centre Growth Reference Study; OFD: Occipito-frontal diameter; SE: Standard error; TCD: Trans cerebellar diameter; UA: Upper arm; LA: Lower arm; WHO: World Health Organization.

## Competing interests

The authors declare that they have no competing interests.

## Authors’ contributions

All authors contributed to the conception and design of the study, read and corrected draft versions of the manuscript and approved the final manuscript.

## Pre-publication history

The pre-publication history for this paper can be accessed here:

http://www.biomedcentral.com/1471-2393/14/157/prepub
